# Randomised feasibility trial to compare three standard of care chemotherapy regimens for early stage triple-negative breast cancer (REaCT-TNBC trial)

**DOI:** 10.1371/journal.pone.0199297

**Published:** 2018-07-24

**Authors:** John Hilton, Carol Stober, Sasha Mazzarello, Lisa Vandermeer, Dean Fergusson, Brian Hutton, Mark Clemons

**Affiliations:** 1 Department of Medicine, Division of Medical Oncology, The Ottawa Hospital and University of Ottawa, Ottawa, Canada; 2 Ottawa Hospital Research Institute and University of Ottawa, Ottawa, Canada; 3 Clinical Epidemiology Program, The Ottawa Hospital Research Institute, Ottawa, Canada; 4 Department of Medicine, The Ottawa Hospital and University of Ottawa School of Epidemiology, Ottawa, Canada; 5 Department of Public Health and Preventative Medicine, The University of Ottawa, Ottawa, Canada; Universita Campus Bio-Medico di Roma, ITALY

## Abstract

**Introduction:**

Despite the importance of chemotherapy in the treatment of early stage triple negative breast cancer (TNBC), no one optimal regimen has been identified. We conducted a pilot trial comparing outcomes for the three most commonly used chemotherapy regimens to assess the feasibility of conducting a larger definitive trial.

**Methods:**

Using integrated consent, newly diagnosed TNBC patients were randomised to one of three standard regimens: dose-dense doxorubicin-cyclophosphamide then paclitaxel, doxorubicin-cyclophosphamide then weekly paclitaxel or 5-FU-epirubicin-cyclophosphamide then docetaxel. Feasibility endpoints included; physician engagement, accrual rates, physician compliance and patient satisfaction with the integrated consent model. Our anticipated pilot trial sample size was 35 randomised patients in one year.

**Results:**

Between August 30^th^, 2016 and January 31^st^ 2017, 2 patients met eligibility and were randomised. A survey of 10 participating oncologists was performed to identify potential strategies to enhance accrual. Most investigators (9/10) believed that the best regimen for TNBC was unknown, and 4/10 felt this was a pressing clinical question. Physicians’ responses suggested that poor accrual was due to: a lack of interest in some study arms as oncologists already had a preferred regimen (4/10) and concerns about trial demands in busy clinics (3/10). The pilot feasibility endpoints were not met and the study was closed.

**Conclusions:**

Despite initial interest in the trial question and multiple investigators agreeing to approach patients, this trial failed to meet feasibility endpoints. The reasons for poor accrual were multiple and require further evaluation if this important patient-centred question is to be answered.

**Trial registration:**

ClinicalTrials.gov NCT02688803.

## Introduction

Triple negative breast cancer (TNBC) accounts for 15–20% of all breast cancers and refers to tumours which have <1% expression of both estrogen (ER) and progesterone receptors (PR) and do not over express human epidermal growth factor receptor 2 (HER2) [1, 2]. Patients diagnosed with TNBC have worse outcomes than patients diagnosed with other types of breast cancer [3]. Despite the poorer prognosis associated with TNBC and the greater relative benefit of chemotherapy [4] in this patient population, the most effective neo/adjuvant chemotherapy regimen has not been identified and confirmed by clinical trial [3, 5–9].

Given that the optimal chemotherapy regimen for TNBC is unknown, we previously surveyed both medical oncologists and patients [10] to assess the feasibility of conducting a clinical trial to address this important question [11]. While the survey confirmed considerable variation in the regimen prescribed between regions and institutions, the most commonly used regimens were: dose dense doxorubicin-cyclophosphamide followed by paclitaxel (ddAC-P), doxorubicin-cyclophosphamide followed by weekly paclitaxel (AC-wP), and 5-FU-epirubicin-cyclophosphamide followed by docetaxel (FEC-D). In addition, both the medical oncologists and patients surveyed expressed enthusiasm and willingness to participate in a clinical trial to identify an optimal chemotherapy regimen.

The challenges of performing randomised trials in an era of costly regulatory burden and low levels of peer-reviewed funding dictate the need for novel pragmatic trials methodologies. We have developed The Rethinking Clinical Trials (REaCT) Program to perform pragmatic clinical trials to compare standard of care interventions [12]. The key components include: demonstration of clinical equipoise through surveys of knowledge users and completion of systematic reviews; simply defined study endpoints and avoidance of superfluous data collection and use of an integrated consent model incorporating oral consent [12]. While the current study incorporated many of these processes, we also needed to demonstrate whether such a methodology and the integrated consent model incorporating oral consent in particular, was feasible for a real-world trial. We therefore conducted a feasibility study assessing whether a pragmatic randomised trial incorporating the integrated consent model could be conducted comparing the three most-commonly used chemotherapy regimens utilized in the curative setting for TNBC [10, 12].

## Materials and methods

### Patients eligibility criteria and enrollment

Patients with newly diagnosed early stage TNBC for whom neo/adjuvant chemotherapy was planned were approached by their medical oncologists at the Ottawa Hospital Cancer Centre and the Irving Greenburg Family Cancer Centre (Ottawa, Ontario, Canada). Eligibility criteria included: histologically confirmed primary TNBC breast cancer, planned for curative neoadjuvant/adjuvant chemotherapy, ≥18 years of age and able to provide verbal consent. Exclusion criteria were either the presence of metastatic disease or a contraindication to any of the chemotherapy agents being evaluated.

### Ethics

The Ottawa Health Science Network Research Ethics Board approval was granted and the trial was registered with clinicaltrials.gov [13]. This study was conducted in accordance with the principles expressed in the Declaration of Helsinki.

### Integrated consent model

Potentially eligible patients were informed about the risks of chemotherapy for the 3 different standard of care regimens. The integrated consent model has been described previously [14], and is akin to a typical conversation between the physician and patient. A copy of the consent template is attached (S1 File). After the patient’s questions were answered, if they were eligible and willing to enter the study, this clinical interaction was documented in the patient’s electronic health record. There was no written consent form and a clinical research associate did not perform the consent process.

### Interventions and randomisation

Eligible and consented patients were randomised using a permuted block design developed by The Ottawa Hospital’s Methods Centre. Randomised patients were stratified by lymph node status and neoadjuvant/adjuvant administration of chemotherapy. Patients were randomised to either:

1. Dose-dense AC-P (doxorubicin 60 mg/m^2^ plus cyclophosphamide 600 mg/m^2^ q2weeks x 4 cycles followed by paclitaxel 175 mg/m^2^ q2 weeks x 4 cycles)
2. Dose-dense AC followed by weekly P (doxorubicin 60 mg/m^2^ plus cyclophosphamide 600 mg/m2 q2weeks x 4 cycles followed by paclitaxel 80 mg/m^2^ weekly x 12 cycles)
3. FEC-D (5-FU 500 mg/m^2^ plus epirubicin 100 mg/m^2^ plus cyclophosphamide 500 mg/m^2^ q3weeks x 3 cycles followed by docetaxel 100 mg/m^2^ q3 weeks x 3 cycles)

### Study endpoints

The primary study objective was to evaluate the feasibility of performing a pragmatic clinical trial comparing different standards for neoadjuvant/adjuvant chemotherapy regimens for patients with TNBC. Feasibility was defined a priori to be considered as successful if: 1) over 50% of appropriate patients approached agreed to participate in the trial; and 2) over 50% of physicians who agreed at study commencement to participate in the study, did indeed approach patients for the study.

Secondary study endpoints of interest included; the proportion of patients who completed their chemotherapy regimen and the frequency of adverse effects requiring hospitalisation/treatment delays (e.g. febrile neutropenia) and were collected from participants medical records and recorded on the case report forms. Adverse events were defined based on the Common Terminology Criteria for Adverse Events (CTCAE) Version 4.0 grading book (National Institute of Health). At the completion of chemotherapy patients also completed a survey to evaluate their perceptions around the REaCT trials process (S2 File).

### Sample size and statistical methods

As this was a feasibility study, there was no a priori planned sample size; however, we anticipated approximately 35 patients would be randomised within one year of study commencement. Data is presented descriptively.

## Results

Obtaining local Research Ethics Board approval for this study took 14 weeks and the trial opened to accrual on August 30^th^, 2016. A total of 8 physicians at both study sites either attended the site initiation visit or completed the on-line trial training. The study was presented twice a month to the breast group at our monthly Academic Breast Rounds and the breast executive meetings. As of January 31^st^, 2017, 2 patients were approached and both enrolled and randomised. One patient was randomised to FEC-D and the other to dose-dense AC-P. None of the approached patients declined study participation. Over the initial six-month study period, only 1 of 8 physicians who agreed at study commencement to participate in the study approached patients for the study it.

**Fig 1:**
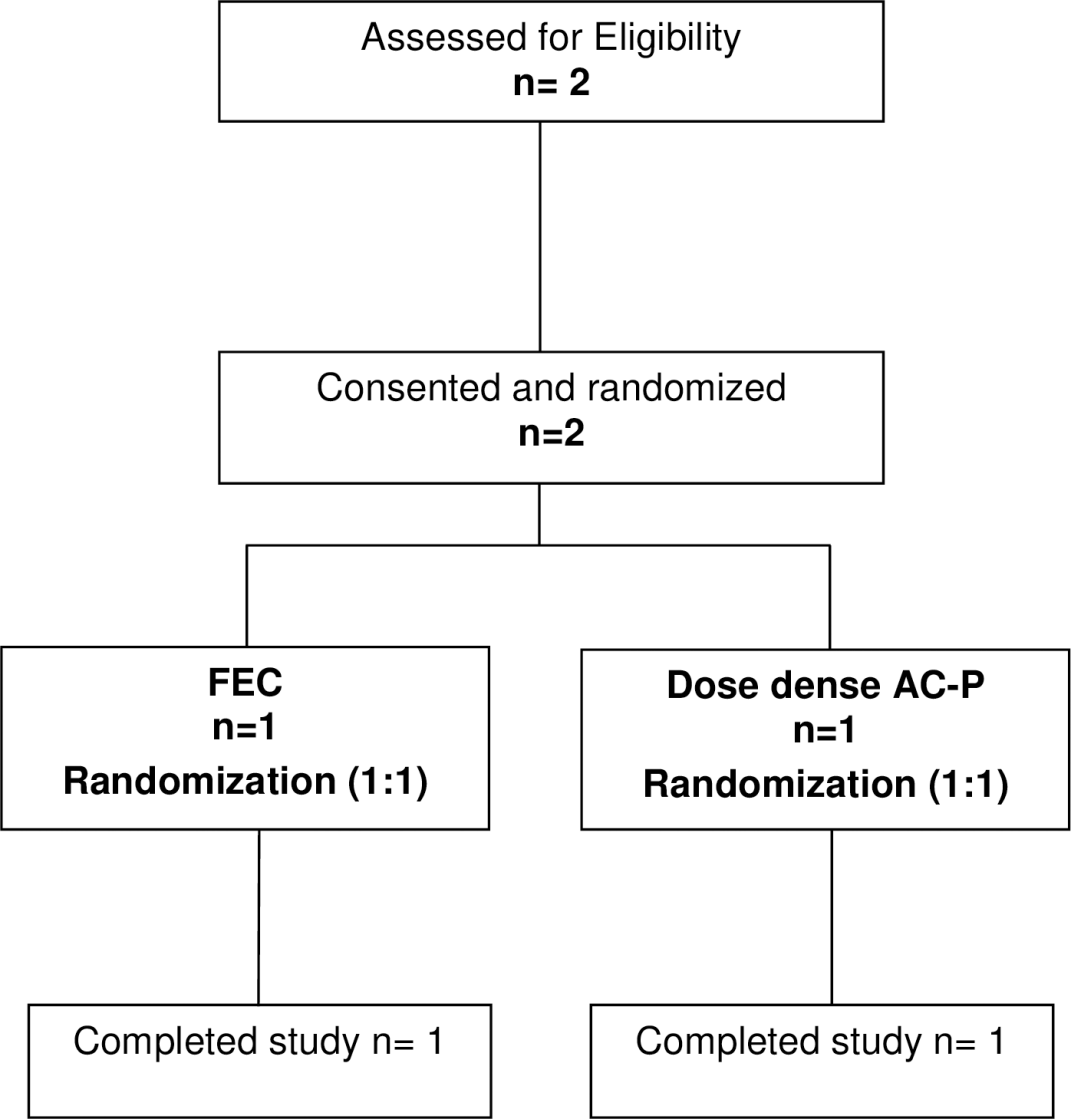
REaCT TNBC CONSORT participant flowchart.

For the secondary endpoints, one patient on AC-P did not complete her chemotherapy due to nausea and taxane-associated pain syndrome [15] while the second patient completed her FEC-D with no complications or significant adverse effects. Neither patient required hospitalization (see Fig 1 and Table 1).

**Table 1:** Patient data.

	Patient 1	Patient 2
Randomised to which chemotherapy regimen?	FEC-D	ddAC-T
Were there any dose adjustments or delays?	Yes	No
Did the participant have treatment-related hospitalization?	No	No
Did the participant complete the study as planned?	Yes	No

### Patient satisfaction survey

Both patients completed their post-chemotherapy satisfaction survey. The main purpose of the survey was to elucidate patient perceptions about the REaCT processes and oral consent. Both patients answered the questions identically, with both agreeing that; “The clinical trial was explained clearly to me by my oncologist”, “I thought that the questions I had about the clinical trial were answered to my satisfaction” and “If I was asked to participate in this study again, I would say yes”. Both patients answered, “disagree” to these questions; “I found that taking part in this study interfered with my quality of life” and “I found that it was time-consuming to take part in this study”.

### Survey of medical oncologists

In order to evaluate the reasons for poor enrollment and to see if there were strategies we could implement to increase accrual, a survey was distributed to 10 medical oncologists treating breast cancer at our centre. These results are presented in Table 2. The median number of new TNBC patients seen a month per physician was 1. Most investigators (9/10) believed that the best regimen for the management of TNBC was unknown, and 4/10 felt this was a pressing clinical question. When asked what clinical questions were more important, 3/10 who responded suggested the need for other targeted agents in combination with chemotherapy. The most common reasons for not approaching patients for the study were identified to be; too few new patients with TNBC (7/10), concerns about clinical trial demands in busy clinics (3/10), lack of interest in some of the study arms as they already had a preferred regimen (4/10), and forgetting about the study (3/10). Thus, despite expressing keen interest to participate prior to study start, there appeared to be little enthusiasm for enrollment. As the feasibility endpoints would not be met, the study was formally closed to accrual on February 8^th^, 2017.

**Table 2:** Result of survey of investigators.

Investigator	Is there good evidence for an optimal chemotherapy treatment for TNBC?	Was the study question of pressing importance to you?	Which topics for TNBC are of greater research importance to you?	How many new TNBC do you see per month?	Why do you think enrollment for this study has gone poorly?
**1**	No	Yes	No reply	2	Trials slow down my clinic; forgot about study
**2**	No	No	Chemotherapy with novel agents	0.5	Already have a preferred regimen
**3**	No	Other topics are of greater interest	Targeted therapies and TNBC subtypes (apocrine, BRCA etc.)	<1	Too few patients have come through my clinic
**4**	No	Other topics are of greater interest	Novel therapies, PARP inhibitors, immunotherapy	3	Less interested in some of the study arms; too few patients
**5**	No	Yes	No reply	<1	Too few patients
**6**	No	Yes	No reply	1	Already have a preferred regimen; too few patients; forgot about study
**7**	No	No	No reply	2	Forgot about study
**8**	Yes	No	Pre-menopausal patients	1	Randomising patients can be challenging; Too few patients
**9**	No	Other topics are of greater interest	Good question, but not interested in participating in a feasibility study	1	Too few patients; Less interested in some of the arms
**10**	No	Yes	No reply	2	Randomising patients can be difficult; Too few potential patients

TNBC = Triple negative breast cancer

## Discussion

Despite the importance of chemotherapy in the management of TNBC, the optimal regimen is unknown. We therefore investigated whether it would be feasible to evaluate the optimal regimen for this disease using novel pragmatic trials methodology. Prior to initiating this study, we conducted a survey of Canadian breast oncologists in order to assess the feasibility of conducting a study to address this question [10]. Based on the overall positive response, our group designed a feasibility study to form the basis to undertake a more definitive study. Unfortunately, after being open for 6 months only two patients were enrolled.

Although it is estimated that around 15% of breast cancer patients are triple negative and given that the participating site historically assesses approximately 800–1000 new patients with breast cancer per year, we did expect a much greater number of potential candidates would be available to be considered for participation. Based on the post-physician survey data, overall volumes of these specific patients were low at around 1 new patient per oncologist per month. Indeed, the oncologists commented that months can go by without seeing a single case of TNBC. Given this situation, it is challenging for any study to develop the necessary momentum to be successful.

Given that enrollment was poor compared to the relative enthusiasm demonstrated in the pre-study survey, we approached participating investigators to better determine why enrollment had had gone poorly. Interestingly, despite the fact that 9/10 acknowledged that we do not have an established optimal chemotherapy regimen for TNBC, 3/10 who responded suggested that they were more interested in novel agents to add to chemotherapy. Given that these novel agents will be added to a standard chemotherapy backbone, determining the optimal chemotherapy regimen should be regarded as a pressing research question. However, most physicians appeared to have minimal enthusiasm about the study overall, and developing a strategy to perform such a study would be challenging [16].

There are clearly limitations to this study. First, only 2 cancer centres were involved and, if more sites had been involved, momentum for the study may have been higher and potentially study feasibility may have been met. Second, physicians we spoke to said that it is challenging to start a conversation with a new patient by trying to explain that that the optimal chemotherapy regimen for their disease is unknown. These physicians felt that it was overall easier and more efficient in a busy clinic just to recommend a given standard of care than offer a clinical trial.

In conclusion, our study failed to meet feasibility and was terminated early. Despite being an important clinical question, we were unable to demonstrate feasibility with the current methodology. The reasons for poor accrual were multiple and require further evaluation if this important clinical question is to be answered.

## Supporting information

S1 File REaCT TNBC consent script. (DOCX)

S2 File REaCT TNBC participant survey. (DOCX)

S3 File REaCT TNB study protocol. (DOCX)

S4 File REaCT TNBC consort checklist. (DOCX)
