# Supplementary material for: Randomised feasibility trial to compare three standard of care chemotherapy regimens for early stage triple-negative breast cancer (REaCT-TNBC trial)
Source: PLoS One. 2018 Jul 24;13(7):e0199297. doi: 10.1371/journal.pone.0199297 (PMC6057636; doi:10.1371/journal.pone.0199297)
Supplement: S1 File — (DOCX) [file pone.0199297.s001.docx]

**Multicentre Study to Determine the Feasibility of using an Integrated Consent Model to Compare Three Standard of Care Regimens for The Treatment of Triple-Negative Breast Cancer in the Neoadjuvant/Adjuvant Setting REaCT-TNBC OTT 15-04**

**Principal Investigator:** [Provincial Investigator’s name and Contact Information]

“Our discussion today is a new approach of informing and consenting patients to participate in this study. The traditional approach is to provide a paper copy of the detailed information sheet and consent form for you to sign. Using this new integrated model for consenting we will have a discussion and you may give a verbal consent to participate or not to participate. I will document our discussion and your decision in your progress note that is part of your health record.

As we've talked about, you will be receiving chemotherapy for your breast cancer. Currently, there are a number of different approved regimens which are used throughout the world to treat your type of breast cancer.

1. Dose dense AC-P (doxorubicin 60 mg/m2 plus cyclophosphamide 600 mg/m2 q2weeks x 4 cycles followed by paclitaxel 175 mg/m2 q2weeks x 4 cycles)

2. Dose dense AC followed by weekly P (doxorubicin 60 mg/m2 plus cyclophosphamide 600 mg/m2 q2weeks x 4 cycles followed by paclitaxel 80 mg/m2 weekly x 12 cycles)

3. FEC-D (5-FU 500 mg/m2 plus epirubicin 100 mg/m2 plus cyclophosphamide 500 mg/m2 q3weeks x 3 cycles followed by docetaxel 100 mg/m2 q3weeks x 3 cycles)

In clinical practice, patients do not switch from regimen to regimen as each one is designed as comprehensive care for TNBC and essentially contain the same backbone (anthracycline/taxane). In addition, in Ontario, an alternative funding source would need to be found as funding is for only the initial primary regimen as per Cancer Care Ontario guidelines. In these very rare situations when there needs to be a switch in therapy, it will be because of a compelling medical reason that prohibits the administration of an anthracycline or taxane. In these situations, patients tend to use non-standard of care regimens not included in the study as determined by their primary oncologist. As these issues are rare and the benefit of switching to a non-standard of care regimen uncertain, this will not be allowed in this study unless patient safety is of issue (this is not different from standard clinical practice).

Because all of these regimens represent standards of care, I can treat you with any one of these different chemotherapy regimens. Because we really don't know if one is better than the other, some of the Oncologists at The Ottawa Hospital Cancer Centre are doing this study by randomly assigning (like pulling a name out of a hat, so that we can obtain an unbiased answer) participants to one of the standards of care and then comparing results over a period of 1 year. We are also looking at how feasible it is for study doctors to enter participants on this study using this integrated consent model. If you choose to participate there won't be any special procedures or visits and you will receive a copy of this document for your reference. If you do participate and decide to stop your participation in the study you may do so but it will not be feasible to switch you to one of the other regimens offered in this study as this is not permitted by Cancer Care Ontario, even if one does not participate in this study. If this occurs, potential treatment options will be discussed with you by your treating oncologist as the options will be very specific to your medical situation.

At the end of the study, there will be a satisfaction survey for you to complete.

There are no additional risks to participating in this study, as all three regimens are standard of care. The Cancer Care Ontario toxicity sheets for the risks for anthracyclines and taxanes are included as appendices.

Your participation in this study is voluntary. If you choose not to participate, your decision will not affect the care you receive at this institution at this time, or in the future. You will not have any penalty or loss of benefits to which you are otherwise entitled to.

You may withdraw from the study at any time without any impact on your current or future care at this institution.

Information that identifies you will be released only if it is required by law. All information collected during your participation in this study will be identified with a unique study number (for example participant # AB01), and will not contain information that identifies you. Documents or samples leaving the Ottawa Hospital Cancer Centre will only contain the coded study number. A Master List provides the link between your identifying information and the coded study number. This list will only be available to [Provincial Investigator’s name] and his staff and will not leave this site. The Master List and coded study records will be stored securely. You will not be identified in any publications or presentations resulting from this study.

All research-related records will be kept for 10 years after termination of the study. No identifiable information will leave this institution. The Ottawa Hospital Science Network Research Ethics Board (OHSN-REB) and the Ottawa Hospital Research Institute may review your relevant study records for audit purposes.

If you have any questions about this study please refer to your list of contact numbers to reach me. The OHSN-REB has reviewed this protocol. The Board considers the ethical aspects of all research studies involving human participants. If you have any questions about your rights as a study participant, you may contact the Chairperson at 613-798-5555, extension 16719

A description of this clinical trial will be available on [*http://www.ClinicalTrials.gov*](http://www.ClinicalTrials.gov).  This website will not include information that can identify you. At most, the website will include a summary of the results. You can search this website at any time.

Do you have any questions?”
