# Supplementary material for: Randomised feasibility trial to compare three standard of care chemotherapy regimens for early stage triple-negative breast cancer (REaCT-TNBC trial)
Source: PLoS One. 2018 Jul 24;13(7):e0199297. doi: 10.1371/journal.pone.0199297 (PMC6057636; doi:10.1371/journal.pone.0199297)
Supplement: S2 File — (DOCX) [file pone.0199297.s002.docx]

**Multicentre Study to Determine the Feasibility of using an Integrated Consent Model to Compare Three Standard of Care Regimens for The Treatment of Triple-Negative Breast Cancer in the Neoadjuvant/Adjuvant Setting (REaCT-TNBC) OTT 15-04**

**Participant Survey**

This participant survey is to be completed at the end of your chemotherapy.

This survey will take approximately 5 minutes to complete, and you may skip any questions that you do not feel comfortable answering. Please use an ink pen when completing the survey.

Thank you for your participation

Participant study # Date:

DD MMM YY

**REaCT OTT 15-04 Participant study #:**

| **Patient Satisfaction Survey** | | | | | | |
| --- | --- | --- | --- | --- | --- | --- |
|  | **Strongly Disagree** | **Disagree** | **Neutral** | **Agree** | **Strongly Agree** | **Not Applicable** |
| The clinical trial was explained clearly to me by my oncologist. |  |  |  |  |  |  |
| I thought that the questions I had about the clinical trial were answered to my satisfaction |  |  |  |  |  |  |
| If I was asked to participate in this study again, I would say yes. |  |  |  |  |  |  |
| I found that taking part in this study interfered with my quality of life. |  |  |  |  |  |  |
| I found that it was time-consuming to take part in this study. |  |  |  |  |  |  |

**Additional comments:**
