# Supplementary material for: Randomised feasibility trial to compare three standard of care chemotherapy regimens for early stage triple-negative breast cancer (REaCT-TNBC trial)
Source: PLoS One. 2018 Jul 24;13(7):e0199297. doi: 10.1371/journal.pone.0199297 (PMC6057636; doi:10.1371/journal.pone.0199297)
Supplement: S3 File — (DOCX) [file pone.0199297.s003.docx]

**Multicentre Study to Determine the Feasibility of using an Integrated Consent Model to Compare Three Standard of Care Regimens for The Treatment of Triple-Negative Breast Cancer in the Neoadjuvant/Adjuvant Setting (REaCT-TNBC) OTT 15-04**

**Coordinating Centre**: The Ottawa Hospital Cancer Centre

**Other Participating Centres:** Kingston General Hospital (Andrew Robinson, PI)

**Qualified Investigator**: John Hilton, MD, FRCPC

The Ottawa Hospital Cancer Centre

501 Smyth Road, Ottawa, Ontario K1H 8L6

Tel: (613) 737-7700 ext. 70179

Fax: (613) 247-3511

E-mail: jfhilton@toh.on.ca

Study Team: Mark Clemons, Sasha Mazzarello, Dean Fergusson, Andrew Robinson (Kingston General Hospital)

**Funding provided by: Internal funding**

Protocol Version May 4, 2016

**PROTOCOL SIGNATURE PAGE**

My signature below confirms that I have reviewed and approved this protocol, and agree that it contains all necessary details for carrying out the study as described. I will conduct this protocol as outlined therein, and according to Good Clinical Practice and all applicable local regulations

Qualified Investigator (Please Print)

Qualified Investigator Signature

Date

Table of Contents

[1 BACKGROUND 4](#_Toc405368666)

[2 OBJECTIVES 7](#_Toc405368667)

[2.1 Primary objective 7](#_Toc405368668)

[2.2 Secondary objective 7](#_Toc405368669)

[3 STUDY DESIGN 7](#_Toc405368670)

[4 METHODS 7](#_Toc405368671)

[5 CONSENT PROCESS 8](#_Toc405368672)

[6 ELIGIBILITY 11](#_Toc405368673)

[6.1 Inclusion Criteria 11](#_Toc405368674)

[6.2 Exclusion Criteria 11](#_Toc405368675)

[7 RANDOMIZATION 11](#_Toc405368676)

[8 DATA COLLECTION 11](#_Toc405368677)

[9 OUTCOMES 12](#_Toc405368678)

[9.1 Primary Outcome 12](#_Toc405368679)

[9.2 Secondary outcome 13](#_Toc405368680)

[10 RISKS 13](#_Toc405368682)

[11 SAFETY REPORTING 13](#_Toc405368683)

[12 MONITORING 13](#_Toc405368684)

[13 CRITERIA FOR FEASABILITY SUCCESS 13](#_Toc405368685)

[14 PREMATURE WITHDRAWAL 14](#_Toc405368687)

[15 SAMPLE SIZE AND STATISTICAL ANALYSIS 14](#_Toc405368688)

[16 References 15](#_Toc405368689)

# BACKGROUND

*Disease of Interest*

Triple-negative breast cancer (TNBC) is a term applied to breast cancer cases that have <1% expression of the estrogen receptor (ER) and the progesterone receptor (PR) and do not over express HER2 [1, 2]. TNBC is diagnosed in 15-20% of breast cancer cases and tends to occur in younger women [3] and have biologically more aggressive high grade disease [4]. Clinically, patients with TNBC have a poorer prognosis compared to patients diagnosed with other breast cancer subtypes [5]. Because of the aggressive phenotype and due to observations that systemic chemotherapy offers significantly higher benefit in ER negative disease, current treatment guidelines from provincial and other organizations [6, 7] recommend that patients receive adjuvant systemic chemotherapy for any TNBC greater than 0.5 cm in greatest diameter or node positive independent of primary tumor size [8].

Currently, there is no world-wide standard recommended chemotherapy regimen for the management of TNBC in the neoadjuvant/adjuvant setting, with treatments varying from region and institution. Although multiple adjuvant regimens have been tested in phase III trials, based on guideline recommendations, three chemotherapy regimens are most frequently prescribed for for TNBC in the province of Ontario:

1. Dose dense AC-P (doxorubicin 60 mg/m2 plus cyclophosphamide 600 mg/m2 q2weeks x 4 cycles followed by paclitaxel 175 mg/m2 q2weeks x 4 cycles) [9]

2. Dose dense AC followed by weekly P (doxorubicin 60 mg/m2 plus cyclophosphamide 600 mg/m2 q2weeks x 4 cycles followed by paclitaxel 80 mg/m2 weekly x 12 cycles) [10]

3. FEC-D (5-FU 500 mg/m2 plus epirubicin 100 mg/m2 plus cyclophosphamide 500 mg/m2 q3weeks x 3 cycles followed by docetaxel 100 mg/m2 q3weeks x 3 cycles) [11]

Although these regimens are widely used and effective, at the current time, they have never been compared head to head in patients with TNBC. Indeed, as all these chemotherapy regimens are funded in Canada, there is no pharmaceutical company interested in funding such a trial. Therefore, there is significant clinical equipoise regarding the best management of these patients. Quite simply **neither patients nor physicians know which is the best of these 3 standard of care regimens to receive for the most aggressive type of breast cancer**.

*Issues Regarding Clinical Trial Design*

When new cancer treatments are developed, they are usually tested in randomized clinical trials (RCTs), where they are directly compared with either a placebo or an established treatment. The results of these usually pharmaceutical company funded trials establish the new treatments as being: better, worse or more commonly equivalent to established therapies. As a result, in clinical practice, physicians may be faced with multiple funded “standards of care”. The reason why comparative standard of care treatment trials are rarely conducted is multifactorial. One of the major barriers is expense. It is incredibly expensive to perform clinical trials as their regulatory oversight (patient consent, REB submission, contracts, drug costs, research coordination and management, data collection of multiple often superfluous endpoints, analysis) has been designed around trials of new agents or established agents for new indications. While these types of studies are also time-consuming, there are sometimes only modest differences between treatment arms. Given that these trials are usually pharmaceutical company funded, they can take many years to start and complete.

As physicians do not know what the “best” treatment for patients is, genuine uncertainty (“clinical equipoise”) exists. Physicians will choose between different “standards” in their personal practice, using idiosyncratic decision making processes, without the physician or the patient knowing the optimal option. This is not good for patients, physicians and society as a whole. Determining the optimal treatment remains an important medical issue for patients, physicians and society. This study will survey opinions on a novel method to allow comparisons of established standard of care prophylactic treatment using the “integrated consent model” as part of a pragmatic clinical trial [12]. By integrating medical and clinical practices, physicians will be able to inform their patients about the RCT, akin to a typical conversation between the physician and patient, without written informed consent. This clinical interaction would then be documented, as ordinarily done in practice, in the patient’s electronic medical record. Medical and clinical practice will be intertwined with the patients’ welfare at the forefront of our best interests.

There is increasing attention being given to pragmatic clinical trials which commonly incorporate comparative effectiveness research – that is, comparing the safety and effectiveness of diagnostic, therapeutic or delivery systems – can help overcome these challenges. These studies, in addition to the ability to leverage patient data from electronic health records to increase sample size of trials at much lower costs, is enabling major national and international initiatives to generate the data needed to improve care [13]. Thus the Integrated Consent Model is being increasingly used internationally to improve patient care [12, 13]. We have already performed a highly successful feasibility study in Ottawa (approved through the local REB) using the Integrated Consent Model to randomise patients receiving TC chemotherapy between G-CSF and ciprofloxacin primary prophylaxis. These are both established and funded standard of care treatments. Feasibility has been demonstrated by successful REB approval, patient and physician engagement in the randomisation process, as well as the collection of results through established hospital datasets. In addition, a second study using the Integrated Consent Model designed by our research team has received provincial IRB approval, demonstrating that this strategy is becoming widely accepted.

This current proposal is similar as all three chemotherapy agents are widely and commonly used with no head to head data. All three consist of an anthracycline (i.e. Adriamycin or epirubin) in combination with cyclophosphamide for 8-12 weeks followed by a taxane (i.e. docetaxel or paclitaxel) for 8 to 12 weeks. The side effects are the same; nausea, vomiting, fatigue, febrile neutropenia, myalgias, mucositis, cardiotoxicity and leukaemia. All are given with standard and anti-emetics for the control of nausea/vomiting as well as concurrent haematopoeitic growth factor support to reduce the risk of febrile neutropenia.

We surveyed oncologists who treat breast cancer at the Ottawa Hospital Cancer Centre. Universally (100% of replies), there was great interest in comparing dose-dense AC-P with FEC-D; or a 3-arm study. This compares well to an informal survey of medical oncologists from across Canada (n=27); when asked to only select one option, 41% would enroll patients on a TNBC study comparing dose-dense AC-P with FEC-D while 44% would prefer a study which evaluated all three of the above regimens (i.e. dose-dense AC-P, FEC-D of Dose dense AC followed by weekly P).

# OBJECTIVES

## Primary objective

To assess the feasibility and physician acceptance of performing a pragmatic multi-centre clinical trial with an Integrated Consent Model for evaluating different standards for neoadjuvant/adjuvant chemotherapy regimens for patients with curable TNBC. Feasibility will be reflected through composite endpoints including; physician engagement, accrual rates, physician compliance and satisfaction, and patient satisfaction.

## Secondary objectives

- Proportion of patients completing all their chemotherapy
- Adverse effects requiring hospitalization/treatment delays (e.g. febrile neutropenia)

# STUDY DESIGN

This will be a multicenter-center open label randomized trial**.**

# METHODS

Investigators will approach potentially eligible breast cancer patients who are planned to receive chemotherapy to treat TNBC in the neoadjuvant/adjuvant setting. The physician will inform their patient about the RCT study, akin to a typical conversation between the physician and patient. Eligible and consented patients will be randomized using a permeated block design developed by The Ottawa Hospital’s Methods Centre to receive one of either:

1. Dose dense AC-P (doxorubicin 60 mg/m2 plus cyclophosphamide 600 mg/m2 q2weeks x 4 cycles followed by paclitaxel 175 mg/m2 q2weeks x 4 cycles) [9]

2. Dose dense AC followed by weekly P (doxorubicin 60 mg/m2 plus cyclophosphamide 600 mg/m2 q2weeks x 4 cycles followed by paclitaxel 80 mg/m2 weekly x 12 cycles) [10]

3. FEC-D (5-FU 500 mg/m2 plus epirubicin 100 mg/m2 plus cyclophosphamide 500 mg/m2 q3weeks x 3 cycles followed by docetaxel 100 mg/m2 q3weeks x 3 cycles) [11]

Patients will be stratified by lymph node status and neoadjuvant/adjuvant administration of chemotherapy.

In clinical practice, patients do not switch from regimen to regimen as each one is designed as comprehensive care for TNBC and essentially contain the same backbone (anthracycline/taxane). In addition, in Ontario, an alternative funding source would need to be found as funding is for only the initial primary regimen as per Cancer Care Ontario guidelines. In these very rare situations when there needs to be a switch in therapy, it will be because of a compelling medical reason that prohibits the administration of an anthracycline or taxane. In these situations, patients tend to use non-standard of care regimens not included in the study as determined by their primary oncologist. As these issues are rare and the benefit of switching to a non-standard of care regimen uncertain, this will not be allowed in this study unless patient safety is of issue (this is not different from standard clinical practice).

# CONSENT PROCESS

In this study the investigator will obtain oral consent using the following prepared REB approved script.

**Multicentre Study to Determine the Feasibility of using an Integrated Consent Model to Compare Three Standard of Care Regimens for The Treatment of Triple-Negative Breast Cancer in the Neoadjuvant/Adjuvant Setting (REaCT-TNBC) OTT 15-04**

“Our discussion today is a new approach of informing and consenting patients to participate in this study. The traditional approach is to provide a paper copy of the detailed information sheet and consent form for you to sign. Using this new integrated model for consenting we will have a discussion and you may give a verbal consent to participate or not to participate. I will document our discussion and your decision in your progress note that is part of your health record.

As we've talked about, you will be receiving chemotherapy for your breast cancer. Currently, there are a number of different approved regimens which are used throughout the world to treat your type of breast cancer.

1. Dose dense AC-P (doxorubicin 60 mg/m2 plus cyclophosphamide 600 mg/m2 q2weeks x 4 cycles followed by paclitaxel 175 mg/m2 q2weeks x 4 cycles)

2. Dose dense AC followed by weekly P (doxorubicin 60 mg/m2 plus cyclophosphamide 600 mg/m2 q2weeks x 4 cycles followed by paclitaxel 80 mg/m2 weekly x 12 cycles)

3. FEC-D (5-FU 500 mg/m2 plus epirubicin 100 mg/m2 plus cyclophosphamide 500 mg/m2 q3weeks x 3 cycles followed by docetaxel 100 mg/m2 q3weeks x 3 cycles)

In clinical practice, patients do not switch from regimen to regimen as each one is designed as comprehensive care for TNBC and essentially contain the same backbone (anthracycline/taxane). In addition, in Ontario, an alternative funding source would need to be found as funding is for only the initial primary regimen as per Cancer Care Ontario guidelines. In these very rare situations when there needs to be a switch in therapy, it will be because of a compelling medical reason that prohibits the administration of an anthracycline or taxane. In these situations, patients tend to use non-standard of care regimens not included in the study as determined by their primary oncologist. As these issues are rare and the benefit of switching to a non-standard of care regimen uncertain, this will not be allowed in this study unless patient safety is of issue (this is not different from standard clinical practice).

Because all of these regimens represent standards of care, I can treat you with any one of these different chemotherapy regimens. Because we really don't know if one is better than the other, some of the Oncologists at The Ottawa Hospital Cancer Centre are doing this study by randomly assigning (like pulling a name out of a hat, so that we can obtain an unbiased answer) participants to one of the standards of care and then comparing results over a period of 1 year. We are also looking at how feasible it is for study doctors to enter participants on this study using this integrated consent model. If you choose to participate there won't be any special procedures or visits and you will receive a copy of this document for your reference. If you do participate and decide to stop your participation in the study you may do so but it will not be feasible to switch you to one of the other regimens offered in this study as this is not permitted by Cancer Care Ontario, even if one does not participate in this study. If this occurs, potential treatment options will be discussed with you by your treating oncologist as the options will be very specific to your medical situation.

At the end of the study, there will be a satisfaction survey for you to complete,

There are no additional risks to participating in this study, as all three regimens are standard of care. The Cancer care Ontario toxicity sheets for the risks for anthracyclines and taxanes are included as appendices.

Your participation in this study is voluntary. If you choose not to participate, your decision will not affect the care you receive at this institution at this time, or in the future. You will not have any penalty or loss of benefits to which you are otherwise entitled to.

You may withdraw from the study at any time without any impact on your current or future care at this institution.

Information that identifies you will be released only if it is required by law. All information collected during your participation in this study will be identified with a unique study number (for example participant # AB01), and will not contain information that identifies you. Documents or samples leaving the Ottawa Hospital Cancer Centre will only contain the coded study number. A Master List provides the link between your identifying information and the coded study number. This list will only be available to Dr. J. Hilton and his staff and will not leave this site. The Master List and coded study records will be stored securely. You will not be identified in any publications or presentations resulting from this study.

All research-related records will be kept for 10 years after termination of the study. No identifiable information will leave this institution. The Ottawa Hospital Science Network Research Ethics Board (OHSN-REB) and the Ottawa Hospital Research Institute, may review your relevant study records for audit purposes.

If you have any questions about this study please refer to your list of contact numbers to reach me. The OHSN-REB has reviewed this protocol. The Board considers the ethical aspects of all research studies involving human participants. If you have any questions about your rights as a study participant, you may contact the Chairperson at 613-798-5555, extension 16719

A description of this clinical trial will be available on [*http://www.ClinicalTrials.gov*](http://www.ClinicalTrials.gov).  This website will not include information that can identify you. At most, the website will include a summary of the results. You can search this website at any time.

Do you have any questions?”

If the patient agrees to participate the physician will dictate in the progress note they have had the above conversation with the patient. There will be no need for the patient to sign an informed consent form.

# ELIGIBILITY

## Inclusion Criteria

- Histologically confirmed primary TNBC breast cancer
- Planned for chemotherapy
- ≥18 years of age
- Able to provide verbal consent

## Exclusion Criteria

- Metastatic disease
- Contraindication to one or more of the chemotherapy agents being evaluated in the study

# RANDOMIZATION

Each site investigator will be able to randomize participants through either their handheld device, their office desk top or by calling the study coordinator (CRA). Each site will have a site code preceding the study number and for each site the study number will be continuous. Patients will be stratified by the goal of therapy (adjuvant or neoadjuvant and presence of lymph nodes). The physician must dictate that the verbal consent and eligibility review has taken place prior to randomization. The investigators will then forward the randomization conformation email indicating the selected study drug to their CRA along with the participant’s medical record number.

# DATA COLLECTION

Once the patient has been randomized, the study CRA will collect participant information treatment assignment, treatment received, and adverse effects requiring hospitalization or treatment delays. This data will all recorded in real-time via electronic health records. From a study standpoint only the CRA will access this information. Data not available at the time of visit will be collected from the dictated physician note. CRAs will complete the paper case report forms (CRF) and for external sites fax the de-identified source documents and CRFs to Dr. Hilton within the timeframes noted on the CRFs. CRFs will also have the contact fax number listed.

Participant data to be collected over the treatment period:

- Treatment assignment
- Treatment received
- Adverse effects requiring hospitalization/treatment delays

If the patient is randomized and either the patient or physician refuses the randomization selection, reasons for this will be recorded. Once assigned to a particular treatment arm, patients will not be permitted to switch to an alternative treatment arm offered in the protocol; this is consistent with Cancer Care Ontario guidelines for treatment standard of care. If physicians choose to break the protocol, they must inform the patient as to why the allocated selection should not be used from the randomization process. We will monitor the charts to ensure that this reason has been recorded. Treatment options at this point would be regarded as non-standard of care and will be left to the discretion of their treating physician. Patients will still be analyzed in the arm that they were assigned despite breaking with protocol treatment (as per intention to treat principles). Patient satisfaction will be assessed using a survey, developed by the study team, following completion of their chemotherapy.

# OUTCOMES

## Primary Outcome

Feasibility of performing this study will be measured with composite endpoints:

1. Percentage of patients who receive chemotherapy in the neoadjuvant/adjuvant setting for TNBC compared to the number of participants who after being approached subsequently agree to randomization.

2. Optional Participant satisfaction survey. Overall participant satisfaction will be determined using the participant survey.

## **Secondary outcome**

1. Percentage of participants who complete study treatment compared to the percentage who discontinue their treatment while on study (compliance) will be calculated using the sites chemotherapy treatment records and data from New Patient Registration.

2. Rate of adverse effects requiring hospitalization/treatment delays

# RISKS

There are no additional risks associated with this study as all arms are standard of care treatments.

# SAFETY REPORTING

For the purpose of this feasibility study SAE reporting is not required. Adverse events specific to the secondary (i.e. hospitalizations) outcome will be captured on the CRFs.

# MONITORING

Monitoring will be performed remotely at The Ottawa Hospital Cancer Centre by their internal monitor for investigator initiated studies. The de-identified source documents and CRFs will be reviewed according to the approved monitoring plan.

# CRITERIA FOR FEASIBILITY SUCCESS

The following criteria were established a priori and needed to be met to deem this feasibility trial successful:

1) Over 50% of appropriate patients approached agree to participate in the RCT,

2) Over 50% of physicians who agree at study commencement to participate in the study do indeed approach patients for the study.

# PREMATURE WITHDRAWAL

Participants have the right to withdraw from the study treatment at any time for any reason

Investigator has the right and obligation to withdraw subjects from the study treatment in the event of:

- Intercurrent illnesses which would, in the judgment of the investigator, affect assessment of clinical status to a significant degree, and require discontinuation of protocol therapy
- Any toxicity that would produce further harm if continued on the protocol
- Request by the participant or of their legally authorized representative (consent withdrawal)
- Non-compliance to the study protocol or logistic consideration
- Participant is lost to follow-up

# SAMPLE SIZE AND STATISTICAL ANALYSIS

As this is a feasibility study there is no pre-defined sample size. We would anticipate that around 35 patients will be entered within one year of study commencement.

Study results will be presented descriptively.

# References

1. Hammond, M.E., et al., *American Society of Clinical Oncology/College of American Pathologists guideline recommendations for immunohistochemical testing of estrogen and progesterone receptors in breast cancer (unabridged version).* Arch Pathol Lab Med, 2010. **134**(7): p. e48-72.

2. Wolff, A.C., et al., *Recommendations for human epidermal growth factor receptor 2 testing in breast cancer: American Society of Clinical Oncology/College of American Pathologists clinical practice guideline update.* J Clin Oncol, 2013. **31**(31): p. 3997-4013.

3. Trivers, K.F., et al., *The epidemiology of triple-negative breast cancer, including race.* Cancer Causes Control, 2009. **20**(7): p. 1071-82.

4. Livasy, C.A., et al., *Phenotypic evaluation of the basal-like subtype of invasive breast carcinoma.* Mod Pathol, 2006. **19**(2): p. 264-71.

5. Lin, N.U., et al., *Clinicopathologic features, patterns of recurrence, and survival among women with triple-negative breast cancer in the National Comprehensive Cancer Network.* Cancer, 2012. **118**(22): p. 5463-72.

6. Gandhi, S., et al., *Adjuvant chemotherapy for early female breast cancer: a systematic review of the evidence for the 2014 Cancer Care Ontario systemic therapy guideline.* Curr Oncol, 2015. **22**: p. S82-94.

7. Gradishar, W.J., et al., *Breast cancer version 3.2014.* J Natl Compr Canc Netw, 2014. **12**(4): p. 542-90.

8. Berry, D.A., et al., *Estrogen-receptor status and outcomes of modern chemotherapy for patients with node-positive breast cancer.* JAMA, 2006. **295**(14): p. 1658-67.

9. Bonilla, L., et al., *Dose-dense chemotherapy in nonmetastatic breast cancer: a systematic review and meta-analysis of randomized controlled trials.* J Natl Cancer Inst, 2010. **102**(24): p. 1845-54.

10. Sparano, J.A., et al., *Weekly paclitaxel in the adjuvant treatment of breast cancer.* N Engl J Med, 2008. **358**(16): p. 1663-71.

11. Roche, H., et al., *Sequential adjuvant epirubicin-based and docetaxel chemotherapy for node-positive breast cancer patients: the FNCLCC PACS 01 Trial.* J Clin Oncol, 2006. **24**(36): p. 5664-71.

12. Kim, S.Y. and F.G. Miller, *Informed consent for pragmatic trials--the integrated consent model.* N Engl J Med, 2014. **370**(8): p. 769-72.

13. Sugarman, J. and R.M. Califf, *Ethics and regulatory complexities for pragmatic clinical trials.* JAMA, 2014. **311**(23): p. 2381-2.
